# Transcriptome analysis of umbilical cord mesenchymal stem cells revealed fetal programming due to chorioamnionitis

**DOI:** 10.1038/s41598-022-10258-0

**Published:** 2022-04-20

**Authors:** Yusuke Noguchi, Atsuko Taki, Izumi Honda, Manabu Sugie, Tsunanori Shidei, Kazuyuki Ito, Haruka Iwata, Akira Koyama, Kaoru Okazaki, Masatoshi Kondo, Chikako Morioka, Kenichi Kashimada, Tomohiro Morio

**Affiliations:** 1 Department of Pediatrics and Developmental Biology, Tokyo Medical and Dental University, 1-5-45 Yushima, Bunkyo-ku, Tokyo, 113-8510 Japan; 2 Department of Obstetrics and Gynecology, Tokyo Metropolitan Tama Medical Center, 2-8-29 Musashidai, Fuchu-shi, Tokyo, 183-8524 Japan; 3 Department of Neonatology, Tokyo Metropolitan Children’s Medical Center, 2-8-29 Musashidai, Fuchu-shi, Tokyo, 183-8561 Japan

**Keywords:** Mesenchymal stem cells, Reprogramming, Stem-cell differentiation, Infection, Neonatal sepsis, Embryonic stem cells, Extracellular matrix, Cell-cycle exit, Cell proliferation

## Abstract

Although chorioamnionitis (CAM) has been demonstrated to be associated with numerous short- and long-term morbidities, the precise mechanisms remain unclear. One of the reasons for this is the lack of appropriate models for analyzing the relationship between the fetal environment and chorioamnionitis and fetal programming in humans. In this study, we aimed to clarify the fetal programming caused by CAM using the gene expression profiles of UCMSCs. From nine preterm neonates with CAM (n = 4) or without CAM (n = 5), we established UCMSCs. The gene expression profiles obtained by RNA-seq analysis revealed distinctive changes in the CAM group USMSCs. The UCMSCs in the CAM group had a myofibroblast-like phenotype with significantly increased expression levels of myofibroblast-related genes, including α-smooth muscle actin (*p* < 0.05). In the pathway analysis, the genes involved in DNA replication and G1 to S cell cycle control were remarkably decreased, suggesting that cellular proliferation was impaired, as confirmed by the cellular proliferation assay (*p* < 0.01–0.05). Pathway analysis revealed that genes related to white fat cell differentiation were significantly increased. Our results could explain the long-term outcomes of patients who were exposed to CAM and revealed that UCMSCs could be an in vitro model of fetal programming affected by CAM.

## Introduction

Fetal programming occurs during embryonic and fetal development, and may cause persistent effects on the fetus and infant long after birth. The concept of fetal programming is derived from the fetal origins hypothesis, that is, Barker’s hypothesis. The concept of fetal programming has established a new approach to identifying the causes of disease, shifting to the in utero environment and its critical role in lifelong health. Fetal programming has been presumed to affect individual gene expression by epigenetic modifications, in which genes are expressed differently without any change to the DNA sequence itself. Further, other biological processes have been supposed to be involved, such as changes in molecular biological functions to permanent hormonal changes, alterations in metabolism, or responses to physiological stressors^1^.

Fetal undernutrition is the most important factor affecting fetal programming, and it has been intensively investigated to date. The fetus adapts to the intrauterine nutritional environment through changes in the regulation of metabolism and redistribution of blood flow, controlling fetal growth^2^. Indeed, the birth weight of babies born after oocyte donation is strongly associated with the weight of the recipient mother rather than the weight of the donor^1^. Fetal nutritional adaptation is explained by the thrifty phenotype hypothesis, and fetal growth restriction has been shown to increase the risk for lifestyle diseases in adults, such as cardiovascular disease, obesity, and diabetes.

Chorioamnionitis is defined as the presence of active infection in the amniotic sac that causes inflammatory changes in the mother. Chorioamnionitis is associated with numerous short-term and long-term morbidities, such as intraventricular hemorrhage, retinopathy of prematurity, chronic lung disease, and cerebral palsy^3^. To date, several studies using animal models have suggested that chorioamnionitis affects fetal programming^4^; however, precise mechanisms are mostly not clarified, especially chorioamnionitis in humans. This is due to the lack of appropriate models for analyzing the relationship between fetal inflammatory environments and fetal programming.

Mesenchymal stem cells (MSCs) have the capacity for proliferation, multilineage differentiation, and immunomodulatory properties, and the properties of cultured MSCs in vitro have been suggested to be applicable for broad medical applications such as regenerative medicine and immunomodulatory medicine against graft versus host disease (GVHD)^5^ and SLE^6^. As a source of MSCs, umbilical cord tissue has been considered a promising option for several reasons. First, the umbilical cord is traditionally regarded as a waste tissue, so isolating MSCs from umbilical cord tissue is not ethically controversial compared with obtaining MSCs from other tissues, such as bone marrow. Second, UCMSCs are mainly located in the subcortical endothelium of the umbilical cord, perivascular region, and Wharton’s jelly (WJ), and can be isolated from umbilical cords by explant monolayer culture, which does not require complicated procedures. Third, in UCMSCs, the expression of human leukocyte antigen (HLA) -ABC and HLA-DR is low, resulting in lower alloreactivity.

In addition to the utilities in regenerative medicine, recent reports suggest that the UCMSCs may be programmed in a manner similar to other MSCs in the fetus, and would be an in vitro model for fetal growth restriction^7^. Analyses of UCMSCs from the neonates born small for gestational age (SGA) were useful in identifying pathways specific to fetal growth restriction^7^. In UCMSCs from neonate with fetal growth restriction, early growth response 1 (EGR1) and cyclooxygenase 2 (Cox2) in the UCMSCs are affected, leading to lower insulin sensitivity and increased adipogenicity^7^.

In the present study, to understand fetal programming caused by CAM, we analyzed UCMSCs obtained from preterm neonates with a history of CAM. Our analysis of USMSCs revealed that UCMCSs could be an in vitro model of fetal programming affected by CAM, providing a tool for clarifying the molecular mechanisms of postnatal complications in neonates who experienced CAM during the fetal period.

## Materials and methods

### Subjects and samples

The present study was approved by the ethical board of Tokyo Medical and Dental University Graduate School of Medicine (M2017-28) and conducted in accordance with the approved guidelines. Written informed consent was obtained from the parents of each neonate. Human umbilical cords were collected from nine very low birth weight infants delivered by cesarean sections at 25–30 weeks of gestation. Clinical data were prospectively collected from the medical records of the neonates and their mothers.

We divided the nine neonates into CAM (n = 4) and non-CAM groups (n = 5). The neonates who exhibited “Triple I,” namely intrauterine inflammation, or infection, or both, were categorized into the CAM group. More specifically, besides pathological findings, maternal fever (> 38.0C), leukocytosis (> 15,000), fetal tachycardia (> 160/min), and definite purulent fluid from the cervical os, were considered as symptoms of CAM^8^. Further, we confirmed CAM by histological analysis of the placenta. On the other hand, with or without identifying histological CAM in placentas, asymptomatic cases were categorized into non- CAM group.

The criterion for chronic lung disease was the requirement of oxygen support at 36 weeks’ corrected postnatal gestational age^9^. Neuromotor development was evaluated according to the Kyoto Scale of Psychological Development 2001, a developmental test that has been widely used by Japanese clinicians working with infants, toddlers, and children. We classified the subjects into three groups based on the TDQ score (normal: > 85, border: 70 ~ 85 and retardation: < 70) (*Shinpan K Shiki Hattatsu Kensahou 2001 Nenban*)^10^.

### Preparation of UCMSCs

Umbilical cord-derived mesenchymal stem cells (UCMSCs) were established according to an improved explant method previously reported^11^. Briefly, a small fragment of the umbilical cord was cultured at 37 °C (5% CO_2_ and 95% air) in MEM-α (Thermo Fisher Scientific, Waltham, MA, USA) with 10% FBS and 2% penicillin–streptomycin (Thermo Fisher Scientific). The outgrowth monolayer cells (Passage1: P1) were collected by disassociating with TrypLE™ Express enzyme (Thermo Fisher Scientific). The collected cells were seeded into the new dishes and frozen stock was collected after reaching confluence (P2). In the present study, we used the cells from the freeze stock (P3).

### RNA extraction

Total RNA from UCMSCs was extracted and purified using the RNeasy Micro Kit (#74106, Qiagen, Hilden, Germany) according to the manufacturer’s instructions. RNA concentration was measured using a Nanodrop ND-8000 spectrophotometer (Nanodrop Technologies, Wilmington, DE, USA).

### Cell surface marker analysis

UCMSCs were dissociated with TrypLE™ Express enzyme (Thermo Fisher Scientific), washed with PBS and suspended. The cells were incubated with phycoeryhrin- (PE-) or Fluoresceinisothiocyanate isomer-I (FITC-) conjugated mouse primary antibodies against CD14, CD19, CD34, CD45, CD73, CD90, CD105, or HLA-DR (BD Bioscience, Franklin Lakes, NJ) for 10 min at room temperature and washed with PBS. Flow cytometry and analysis was performed using BD LSRFortessa™, FACSDiva software and FlowJo™ software (BD Bioscience).

### RNA seq

#### Library preparation and sequencing

The sequencing libraries from total RNA of USMSC were constructed using the NEBNext Ultra II Directional RNA Library Prep Kit for Illumina (#E7760, New England Biolabs, MA, USA) with NEBNext Poly(A) mRNA Magnetic Isolation Module according to the manufacturer’s protocols. The quality of the libraries was assessed using an Agilent 2200 TapeStation High Sensitivity D1000 (Agilent Technologies, Inc., Santa Clara, CA, USA). The pooled libraries of the samples were sequenced using the Illumina NextSeq 500 (Illumina, Inc., San Diego, CA, USA) in 76-base-pair (bp) single-end reads.

#### Alignment to the whole transcriptome

Sequencing adaptors, low quality reads, and bases were trimmed using the Trimmomatic-0.38 tool^12^. The sequence reads were aligned to the human reference genome (hg19) using STAR 2.7^13^. For the whole transcriptome alignment with the STAR, files of the gene model annotations and known transcripts were downloaded from the Illumina’s iGenomes website (http://support.illumina.com/sequencing/sequencing_software/igenome.html).

#### Quantifying the gene expression levels and detection of differentially expressed genes

The aligned reads were subjected to downstream analyses using StrandNGS 3.2 software (Agilent Technologies). The read counts allocated for each gene and transcript RefSeq Genes (2015.10.05) were quantified using the trimmed mean of M-value (TMM) method^14^. To investigate gene expression differences, we selected genes through moderated t-test (Benjamini–Hochberg multiple test correction FDR-q-value < 0.05) and up- or downregulated them by setting a threshold of twofold. To summarize the biological aspects of the selected genes, we employed a volcano plot, Gene Ontology (GO) terms, and pathway analysis.

For the volcano plot, the genes of each category were selected using the following procedures. The representative genes involved in the contractile apparatus and extracellular matrix were selected based on previous reports^15–18^. We selected cell cycle genes that annotated GO:0045787 positive regulation of cell cycle, fold change < 0.5, and *p* < 0.05, or that annotated GO:0045786 negative regulation of cell cycle, fold change > 2, and *p* < 0.05. We used R software version 4.1.1 (R-Tools Technology Inc., ON, Canada) for statistical analysis.

Pathway statistical analysis was performed on a pathway collection of the WikiPathways^19^ database using PathVisio tool^20^ to determine pathways containing the most changed expression, taking into consideration the number of genes in the pathway that were measured in the experiment and the number of genes that were differentially expressed.

### Quantitative real-time PCR

cDNA was synthesized from 800 ng of total RNA from UCMSCs by using a High Capacity cDNA Reverse Transcription Kit (#4368814, Thermo). Real-time PCR analysis was performed with a Roche Lightcycler 480II real-time PCR system (Roche Diagnostics, Mannheim, Germany) using FastStart Universal SYBR Green master mix (#4913914001, Roche) with 0.5 μM sense and antisense primers and cDNA (corresponding to 25 ng total RNA) according to the manufacturer’s instructions. The relative expression of each transcript was calculated based on the calibration curve method using GAPDH as an endogenous reference for normalization. The primer sets are listed in Table S2. Biologically independent (n = 4 or 5) experiments were performed, and all sample measurements were repeated at least three times.

### Cell proliferation assay

UCMSCs were seeded at the density of 1 × 10^6^ cells per 10 cm cell culture dish. The cells were passaged three times every 2 days and the number of the cells at each passage was counted. Biologically independent (n = 4 or 5) experiments were performed, and all sample measurements were repeated at least twice.

### MTS assay

At the density of 1 × 10^4^ cells per 96 well plate, we seeded UCMCSs, and the cells were incubated at 37 °C (5% CO_2_ and 95% air) for 24 h. Cell proliferation was measured by the CellTiter 96^®^ AQ_ueous_ One Solution Cell Proliferation Assay kit (#G3582, Promega, Madison, WI, USA) according to the manufacturer’s instruction. Briefly, 20 μl of MTS reagent (a tetrazolium compound and an electron coupling reagent) was added into each well and incubated at 37 °C (5% CO_2_ and 95% air) for 4 h. The absorbance at 450 nm was measured using an iMark™ Microplate Reader (#168-1130JA, BIO-RAD Laboratories, Inc, Hercules, CA). Biologically three independent experiments were performed, and all sample were measured with five replicates. We calculated the average and SE of each sample.

### Cell cycle analysis

UCMSCs were seeded at 6 × 10^5^ cells/per 10 cm dish and incubated at 37 °C (5% CO_2_ and 95% air) for 24 h. At 60–70% confluence, cells were performed cell cycle analysis. Briefly, cells were washed and fixed in 70% ethanol for 2 h at – 20 °C. Fixed cells were washed and incubated in 0.25 mg/ml RNase A (#12091039, Thermo Fisher Science) for 30 min at 37 °C. Subsequently, cells were stained with 50 μg/ml propidium iodide (PI) (#25535-16-4, BioVison, Inc, Milpitas, CA) for 30 min at 4 °C in the dark. Cell cycles were assessed by flow cytometry and analysis was performed using BD LSRFortessa™, FACSDiva software and FlowJo™ software (BD Bioscience), and counted the number of the cells at each cell cycle phase.

### Statistical analysis

Real-time PCR was analyzed using the Mann–Whitney *U* test. Student’s *t* test was used for cell proliferation and cell cycle analyses. Clinical data and experimental data were compared using Fisher’s exact test, Mann–Whitney *U* test, or Student’s *t* test, as required. Cell cycle analysis.

For statistical analysis, we used JMP Pro version 15.1.0 (SAS Institute Inc, NC, USA). Statistical significance was set at *p* < 0.05. Significant differences were expressed as ‘*’ for *P* values, 0.05, ‘**’ for *P* values ,0.01 and ‘***’ for P-values ,0.001 respectively.

## Results

### The UCMSCs would be an in vitro model of fetal programming affected by CAM

We obtained the cells from umbilical cords of neonates with CAM (n = 4) and normal healthy controls (n = 5) by explant monolayer culture. There were no significant differences in the clinical backgrounds between the normal healthy group and the CAM group, except for the pathological grade of chorioamnionitis and funisitis (Table 1). No significant differences in the frequency of CLD and neurodevelopment until 3 years old between the two groups (Supplementary Table 1) were observed. We selected three samples from each group and performed transcriptome analysis.

**Table 1:** Clinical backgrounds of the subjects.

	Control	CAM	*p*
n	5	4	
Gestational age (weeks)^†^	28.0 ± 1.0	27.9 ± 1.1	1
Birth weight (g)^†^	1056 ± 94	1157 ± 107	0.556
Sex (Male/Female)	2/3	2/2	0.764
Umbilical artery pH^†^	7.31 ± 0.06	7.35 ± 0.04	1
Clinical Chorioamnionitis	0 (0)	4 (100)	0.003*
Pathological Chorioamnionitis higher than grade 2 (%)	0 (0)	4 (100)	0.003*
Pathological Funisitis (%)	0 (0)	2 (50)	0.073

*p < 0.05, ^†^average ± SE.

**Figure 1:**
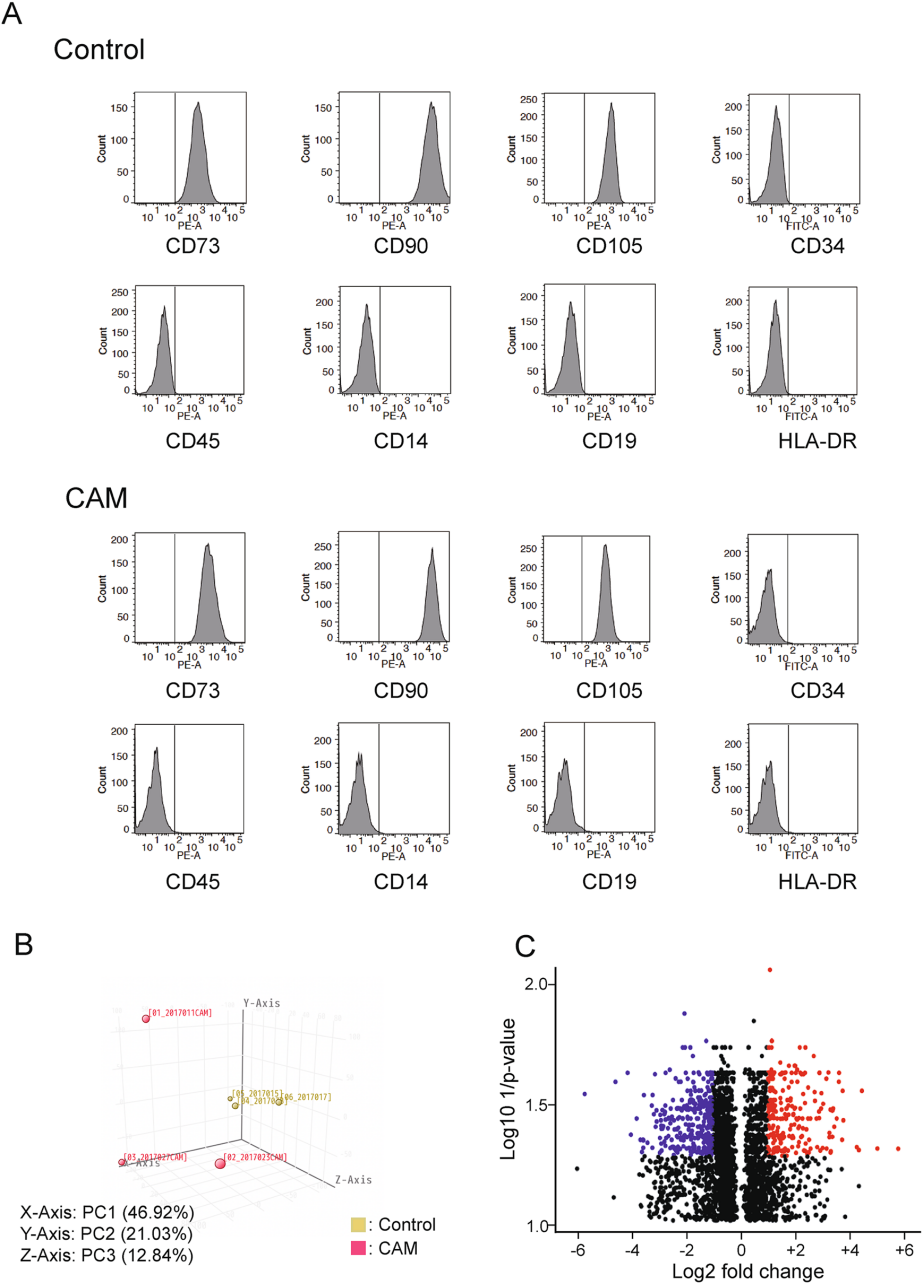
(**A**): Surface marker analysis by flow cytometry revealed that the cells obtained from umbilical cords had UCMSC features. The cells expressed CD73, CD90, and CD105, while not CD34, CD45, CD14, CD19, and HLA-DR; (**B**): Principal component analysis revealed that the gene expression profiles of the CAM group (red) were distinctively different from the normal healthy control, non-CAM group (yellow). (**C**): Volcano plot displaying the results of RNA-seq results from the CAM group and normal healthy controls. The genes that passed the thresholds for *p* value (Log10 1/*p* value > 1.30) and log fold change (Log2 fold change > + 1 or < −1) were colored (red for upregulated and blue for downregulated).

The collected cells from umbilical cords highly expressed CD73, CD90 and CD105, while the expression of CD34, CD45, CD14, CD19, and HLA-DR were low, indicating that the cells had the features of UCMSCs (Fig. 1A, Fig. S1). On the other hand, principal component analysis revealed that the gene expression profiles were distinctively changed in the CAM group (Fig. 1B), and a substantial number of genes were significantly upregulated or downregulated (Fig. 1C, Table 2).

**Table 2:** (1) The top 20 upregulated Genes in the CAM group, (2) The top 20 downregulated Genes in the CAM group.

Gene ID	Gene symbol	Description	FC	*p*	FDR
**(1)**					
11185	INMT	Indolethylamine *N*-methyltransferase	55.75	0.00146089	0.04660115
56892	C8orf4	Chromosome 8 open reading frame 4	32.95	0.00144972	0.04651951
2359	FPR3	Formyl peptide receptor 3	22.16	1.60E−04	0.02638678
55806	HR	Hair growth associated	20.58	0.00150932	0.04717533
953	ENTPD1	Ectonucleoside triphosphate diphosphohydrolase 1	19.47	0.00135167	0.04586875
339855	KY	Kyphoscoliosis peptidase	13.83	6.23E−04	0.03525798
7038	TG	thyroglobulin	13.58	1.66E−04	0.02638678
94122	SYTL5	Synaptotagmin-like 5	13.40	0.00115483	0.04265808
4909	NTF4	Neurotrophin 4	12.42	5.16E−05	0.02211129
402778	IFITM10	interferon induced transmembrane protein 10	12.35	1.02E−04	0.02413612
81285	OR51E2	Olfactory receptor, family 51, subfamily E, member 2	11.69	5.74E−04	0.03463629
343450	KCNT2	potassium channel, subfamily T, member 2	10.97	7.41E−04	0.03756413
4239	MFAP4	Microfibrillar-associated protein 4	10.68	3.81E−04	0.03140028
6649	SOD3	Superoxide dismutase 3, extracellular	10.47	4.36E−04	0.03202961
92973	LINC00950	Long intergenic non-protein coding RNA 950	10.46	0.0010288	0.0414545
22801	ITGA11	Integrin, alpha 11	10.10	4.12E−04	0.03174352
348	APOE	Apolipoprotein E	10.08	0.00158066	0.04778386
775	CACNA1C	Calcium channel, voltage-dependent, L type, alpha 1C subunit	10.01	0.00164752	0.04833471
1066	CES1	carboxylesterase 1	10.00	4.10E−04	0.03174352
619279	ZNF704	Zinc finger protein 704	9.72	8.78E−04	0.03985729
**(2)**					
119	ADD2	Adducin 2 (beta)	51.90	1.84E−04	0.02722342
11144	DMC1	DNA meiotic recombinase 1	23.71	1.11E−04	0.02415509
1870	E2F2	E2F transcription factor 2	17.45	3.29E−05	0.02211129
64641	EBF2	early B-cell factor 2	16.02	9.23E−04	0.04057951
374393	FAM111B	Family with sequence similarity 111, member B	13.87	5.88E−04	0.03463629
4998	ORC1	Origin recognition complex, subunit 1	12.61	0.00114131	0.04265808
1364	CLDN4	Claudin 4	12.12	0.00160917	0.04807075
195828	ZNF367	Zinc finger protein 367	11.78	0.00114501	0.04265808
9088	PKMYT1	Protein kinase, membrane associated tyrosine/threonine 1	11.70	0.0013774	0.04616627
**9134**	**CCNE2**	**Cyclin E2**	**11.68**	**0.00117495**	**0.04287682**
114898	C1QTNF2	C1q and tumor necrosis factor related protein 2	11.50	1.93E−04	0.02752812
**10721**	**POLQ**	Polymerase (DNA directed), theta	10.52	0.00124078	0.04389183
51659	GINS2	GINS complex subunit 2 (Psf2 homolog)	9.43	3.12E−04	0.03050493
8438	RAD54L	RAD54-like (S. cerevisiae)	9.29	0.00138905	0.04616627
81620	CDT1	Chromatin licensing and DNA replication factor 1	9.23	1.91E−04	0.02749984
84125	LRRIQ1	Leucine-rich repeats and IQ motif containing 1	8.93	4.78E−04	0.03318961
**993**	**CDC25A**	**Cell division cycle 25A**	**8.69**	**7.08E−05**	**0.02241816**
**8900**	**CCNA1**	**cyclin A1**	**8.56**	**7.39E−04**	**0.03756413**
10635	RAD51AP1	RAD51 associated protein 1	8.37	4.34E−04	0.03197279
**79019**	**CENPM**	**Centromere protein M**	**8.33**	**6.39E−04**	**0.03568164**

Bold: Cell cycle related genes.

### The features of the gene expression profile of UCMSCs in the CAM group

#### Myofibroblast-like profiles

**Figure 2:**
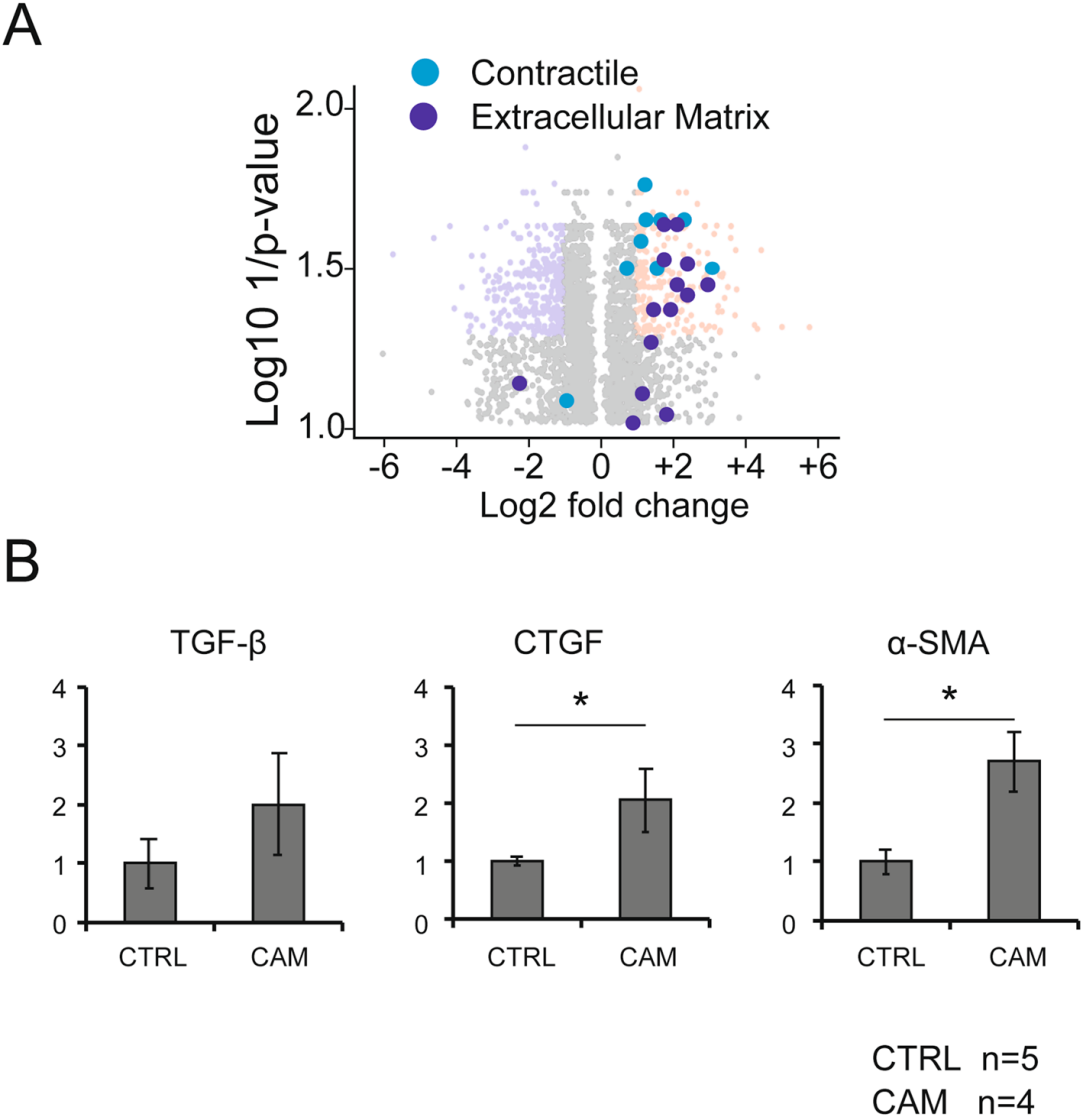
(**A**): Genes related to the contractile apparatus (light blue) and extracellular matrix (deep blue), were plotted on the volcano plot. (**B**): Real time analysis of the myofibroblast marker genes, the CAM group: n = 4, and the non CAM group (CTRL): n = 5, *p < 0.05, Error bars indicate ± SE, Mann–Whitney analysis was used for statistical analysis.

Gene ontology analyses revealed that the genes annotated to extracellular structure, collagen catabolic process and collagen metabolic process were remarkably activated (Table 3). Furthermore, pathway analysis showed that the genes associated with focal adhesion, striated muscle contraction, and human primary endometrial stromal cells were significantly upregulated (Table 4). This observation supported the data from the gene expression profile in which the myofibroblast-related genes were upregulated (Fig. 2A). Quantitative RT-PCR analysis confirmed that the expression level of the myofibroblast marker, α-smooth muscle actin (α-SMA) was significantly increased (Fig. 2B), suggesting that the phenotypes of UCMSCs with CAM would be shifted to the that of myofibroblasts.

**Table 3:** 1 Over-represented Gene Ontology Classes, 2 Under-represented Gene Ontology Classes.

GO term	Count in selection	*p* value	FDR
**(1)**			
Biological Process			
*Extracellular structure organization	20	5.112E−10	3.400E−06
*Extracellular matrix organization	19	6.497E−10	3.457E−06
*Collagen catabolic process	10	4.219E−09	1.403E−05
*Collagen metabolic process	10	1.564E−08	4.161E−05
*Extracellular matrix disassembly	9	1.694E−07	3.757E−04
^†^Cell cycle arrest	10	2.125E−06	3.141E−03
*Collagen fibril organization	6	2.940E−06	3.910E−03
Response to acid chemical	12	1.094E−05	1.120E−02
Response to toxic substance	13	4.999E−05	4.290E−02
Autophagy	7	9.546E−05	7.055E−02
Process utilizing autophagic mechanism	7	9.546E−05	7.055E−02
Autophagosome assembly	4	1.157E−04	7.327E−02
Autophagosome organization	4	1.157E−04	7.327E−02
Cellular component disassembly	13	1.059E−04	7.327E−02
Regulation of growth	16	1.648E−04	9.963E−02
Molecular function			
*Extracellular matrix structural constituent	10	1.384E−08	4.090E−05
Growth factor binding	9	2.872E−06	3.910E−03
*Platelet-derived growth factor binding	4	4.007E−06	5.077E−03
**(2)**			
Biological Process			
DNA metabolic process	80	0.000E + 00	0.000E + 00
^†^Cell cycle	103	0.000E + 00	0.000E + 00
^†^Mitotic cell cycle	82	0.000E + 00	0.000E + 00
^†^DNA replication	45	3.309E−39	1.775E−35
^†^Cell cycle process	78	2.239E−38	9.004E−35
^†^Mitotic cell cycle process	58	1.619E−33	4.341E−30
^†^DNA-dependent DNA replication	28	4.829E−30	1.110E−26
DNA repair	50	1.065E−29	2.285E−26
^†^DNA strand elongation involved in DNA replication	20	7.749E−28	1.558E−24
Chromosome organization	64	1.725E−27	3.264E−24
Molecular function			
Protein binding	188	9.834E−13	5.455E−10
Catalytic activity, acting on DNA	20	6.426E−12	3.335E−09
Nucleic acid binding	104	1.667E−11	8.380E−09
Heterocyclic compound binding	140	2.120E−10	9.745E−08
Organic cyclic compound binding	141	2.508E−10	1.136E−07
Nucleotide binding	77	1.431E−09	5.757E−07
Nucleoside phosphate binding	77	1.460E−09	5.799E−07
DNA−dependent ATPase activity	12	2.105E−09	8.064E−07
DNA binding	77	4.162E−09	1.456E−06
Nucleoside−triphosphatase activity	37	6.910E−09	2.340E−06

*Related to myofibroblast differentiation.

^†^Related to cell cycle.

**Table 4:** Statistically significant pathways.

Pathway	Pathway entities	Matched entities	Fold change	*p* value
***Myofibroblast***				
Focal Adhesion	206	39	4.80	0.000
Striated Muscle Contraction	38	13	5.29	0.001
BMP2-WNT4-FOXO1 pathway in Human Primary Endometrial Stromal Cell	13	4	2.66	0.014

Pathway	Pathway entities	Matched entities	Fold change	*p* value
***Cell Cycle***				
DNA Replication	41	29	− 14.95	0.000
G1 to S cell cycle control	64	28	− 10.67	0.000
Cell cycle	120	39	− 10.06	0.000
ATM Signaling Pathway	41	12	− 5.08	0.000

Pathway	Pathway entities	Matched entities	Fold change	*p* value
***Adipogenesis***				
Transcription factor regulation in Adipogenesis	22	8	4.37	0.001
Adipogenesis	131	25	3.87	0.001
White fat cell differentiation	32	8	3.06	0.008
Leptin Insulin Overlap	17	4	2.02	0.039

#### Inhibition of the cell cycle

**Figure 3:**
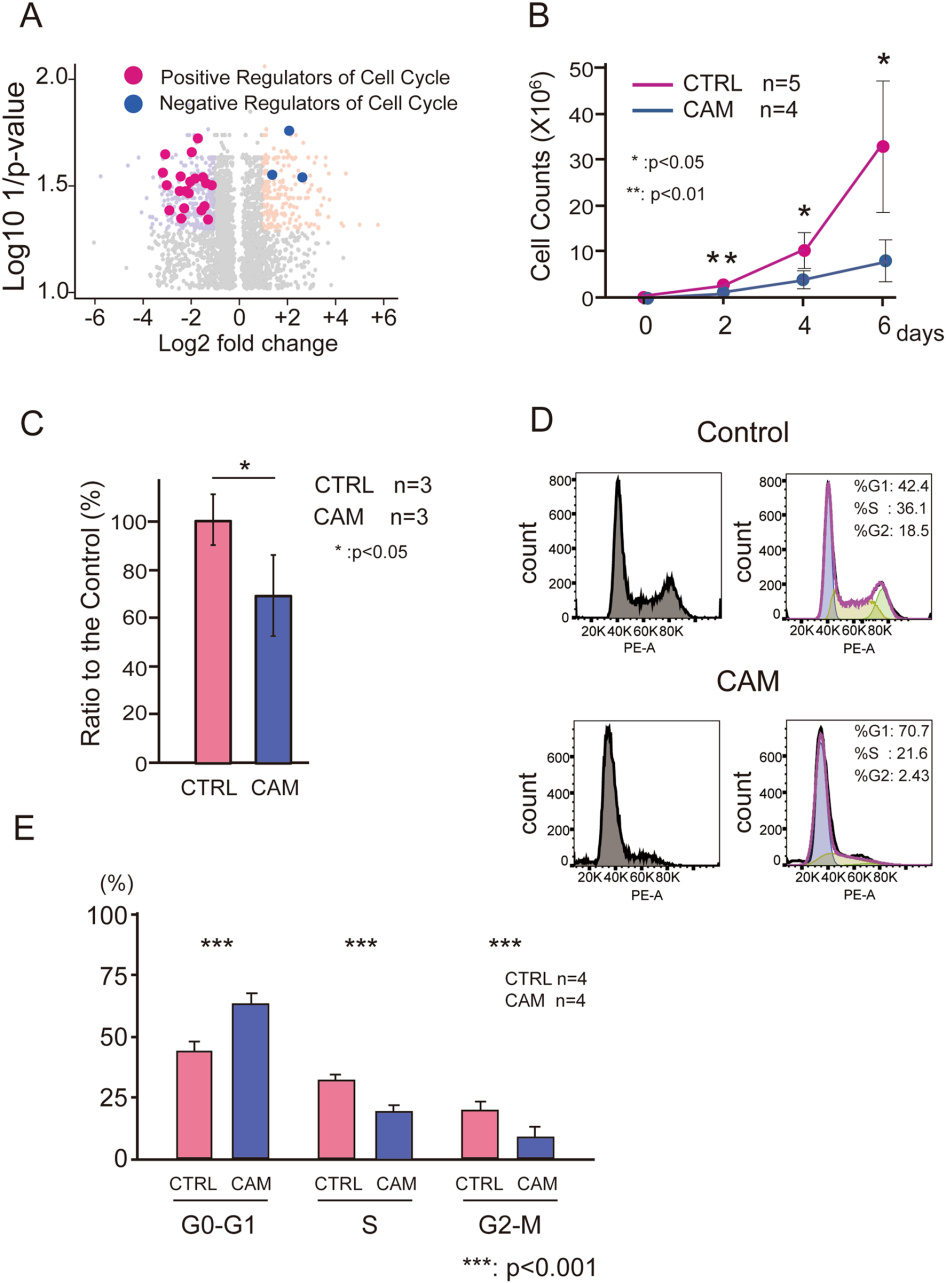
(**A**): Genes related positive regulation of cell cycle (red), and negative regulation of cell cycle (blue) were plotted on the volcano plot. (**B**): Cell proliferation assay. We passaged the cells three times every 2 days (days 2, 4, and 6) and counted the number of the cells at each passage. (control: n = 5, CAM: n = 4) *p < 0.05, **p < 0.01, Error bars indicate ± SE. Student’s *t* test was used for statistical analysis. (**C**): MTS assay of UCMSCs statistically revealed the significant difference between the control (n = 3) and the CAM group (n = 3). *p < 0.05, Error bars indicate ± SE. Student’s *t *test was used for statistical analysis. (**D**): Representative data of the cell cycle distribution of the UCMSCs from the control and the CAM group. Compared with the control group, the cells in G2/S phase were decreased in the CAM group. (**E**): A comparison of the UCMSCs population at each phase of the cell cycle revealed the significant difference between the control (n = 4) and the CAM group (n = 4), ***p < 0.001, Error bars indicate ± SE. Student’s *t* test was used for statistical analysis.

The cell cycle of UCMSCs from the CAM group was significantly suppressed (Table 3). The downregulation of the genes annotated to activate cell cycles, such as cell cycle, mitotic cell cycle, DNA replication, DNA-dependent DNA replication, etc., were statistically significant (Fig. 3A, Table 3). The cell cycle suppressor genes, including genes involved in cell cycle arrest, were activated (Fig. 3A, Table 3). Pathway analysis revealed that the genes involved in DNA replication, G1 to S cell cycle control, cell cycle, and ATM signaling pathway were remarkably decreased (Table 4). Consistently, the proliferation of UCMSCs in the CAM group was significantly impaired (Fig. 3B,C), and the population in the synthesis (S), and the second growth (G2) phases were reduced in the CAM group (Fig. 3D,E).

#### Activated adipogenesis related genes

In pathway analysis, the genes related to adipogenesis, including transcription factor regulation in adipogenesis, white fat cell differentiation, and leptin insulin overlap were significantly increased (Table 4). In contrast, brown fat cell differentiation related genes did not change significantly (data not shown). This observation suggests that CAM during the fetal period selectively activates the genes that contribute to the differentiation of white fat cells.

## Discussion

Our analyses revealed that a fetal environment with CAM affected the characteristics of MSCs with dramatically altered gene expression profiles, promoting myofibroblastic and white adipocyte differentiation with reduced capability for cell proliferation. Our present study highlights three points: (a) UCMSCs would be an excellent model that reflect the fetal programming due to CAM, (b) the affected gene expression profiles of UCMSC by CAM could explain the postnatal complications of neonates who experienced CAM during the fetal period, and (c) for future utilization of autologous cell treatment, CAM would affect the outcomes of the therapy.

Our analysis revealed that the genetic expression profiles of UCMSC were profoundly deviated by exposure to CAM, suggesting that CAM would cause fetal programming to affect the long- term outcomes of offspring. In the context of fetal programming, extensive research has focused on maternal nutritional status and later metabolic disease in neonates, and CAM has been a sub-focus. Indeed, the major adverse outcomes due to CAM, such as sepsis and neurodevelopmental problems, are mainly caused by the direct effects of infection or inflammatory injury due to cytokines^21,22^. However, in the last decade, the placenta has become a new focus in fetal programming research^23^. If placental function, which underpins fetal development, is impaired, fetal development may be compromised. Historically, one of the most striking fetal programming findings come from examination of the subjects who were exposed to the 1918 (Spanish) influenza pandemic in utero^24^. Accordingly, it is not surprising that CAM, a condition of placental inflammation, would lead to compromised fetal programming. Additionally, we consider the possibility that infection or inflammatory injury could be another cause for fetal programming. For clarifying the possibility, further accumulation of data from the cases and disease models are required.

Recently, programmed differences in UCMSCs have been reported to reflect the effects of a maternal metabolic environment, and UCMSC is a model of fetal programming^7,25^. One of the peculiar characteristics of UCMCSs from CAM neonates was a myofibroblast-like phenotype. Myofibroblasts are contractile, α-smooth muscle actin-positive cells with multiple roles in pathophysiological processes including mediating wound contraction^26^. The persistent presence of myofibroblasts in tissues promotes tissue fibrosis. CLD, also known as bronchopulmonary dysplasia (BPD), is the most common chronic respiratory disorder in preterm infants and is characterized by an interruption in pulmonary vascular and alveolar development^27^. The etiology of CLD is multifactorial and involves antenatal and/or postnatal factors, such as intrauterine growth restriction, maternal smoking, mechanical ventilation, oxygen toxicity, and infection, which impair lung maturation. Although it is not conclusive, some studies have revealed an association between CAM and CLD^8,28–30^, and MSC differentiation into myofibroblasts is likely to be involved in the pathophysiology of CLD^17^. Our data, the myofibroblastic phenotypes of UCMSCs may explain the risk of CLD in neonates with CAM.

Our analysis found that s from CAM patients highly expressed white adipocyte related genes, suggesting that CAM alters the programmed gene expression profile in MSC metabolism. As previously observed in the influence of maternal metabolic environment on the fetal metabolome and genome in^7,31^, CAM also might cause programmed differences in stem cell metabolism, which could lead to differences in body composition in later life stages. Although the association between CAM and body composition or lipid metabolism in later life has not been clarified, prenatal inflammation of the placenta would cause metabolic disorders, as described in cases of exposure to the 1918 influenza pandemic during the fetal period^32^.

The proliferative ability of UCMSCs was profoundly affected by CAM. This suggests that the proliferative ability of USMSCs is vulnerable to the inflammatory environment during the fetal period. MSCs are thought to directly respond to inflammatory stimuli by cytokine priming, leading to acquiring their anti-inflammatory and immunomodulatory activities in situ^33–35^. The anti-inflammatory and immunomodulatory effects can occur through cellular contact and/or the secretion of diverse factors^36^, and the characteristics of MSCs enable immune modulation and anti-inflammatory applications that are broadly applicable in damaged tissue. Although, the significance of cellular growth for the therapeutic applications has not been clarified, our data suggest that inflammatory stimuli during the fetal period potentially affect the outcomes of therapeutic applications with UCMSCs. In addition to UCMSCs, the profiles of other neonate derived MSCs, such as cord-blood derived MSCs, could be affected by inflammatory stimuli during the fetal period. Based on our findings, further careful evaluation of the influence of inflammatory stimuli on neonate derived MSCs is required.

The present study has some limitations. Our findings are mainly based on the gene expression profiles of UCMSCs, and the biological and clinical relevance of these findings have not been clarified in detail. Despite this limitation, we presume that UCMSCs is a potential mode of the fetal programming caused by CAM. The characteristics of the gene expression profiles in UCMSCs from CAM neonates could explain the pathophysiology of the complications due to CAM, such as chronic lung disease (CLD). Long-term follow-up and trajectory analysis of CAM cases with a large cohort is essential to elucidate the biological and clinical relevance of UCMSC as a model of a fetal inflammatory environment.

In summary, our data revealed that UCMSC would be an excellent model that reflects fetal programming, and CAM is another factor that causes fetal programming, affecting the long-term outcomes of offspring. Our findings would provide valuable insights for understanding fetal programming caused by CAM and the development of optimal protocols for the applications of regenerative medicine using USMSCs.

## Supplementary Information

Supplementary Figure Legend.

Supplementary Figure S1.

Supplementary Table S1.

Supplementary Table S2.

Supplementary Table S3.
