# Supplementary material for: Transcriptome analysis of umbilical cord mesenchymal stem cells revealed fetal programming due to chorioamnionitis
Source: Sci Rep. 2022 Apr 20;12:6537. doi: 10.1038/s41598-022-10258-0 (PMC9021264; doi:10.1038/s41598-022-10258-0)
Supplement: Supplementary file 1 — Supplementary Figure Legend. [file 41598_2022_10258_MOESM1_ESM.docx]

**Supplementary information**

**Figure S1**

RNA-seq analysis confirmed that the cells obtained from umbilical cords had UCMSC features, i.e., high mRNA expression of CD73, CD90, and CD105, while low expression levels of CD34, CD45, CD11b CD19, and HLA-DR. White bars: normal healthy control (non-CAM); gray bars: CAM; error bars indicate +/-SE
