# Supplementary figures and images for: Transcriptome analysis of umbilical cord mesenchymal stem cells revealed fetal programming due to chorioamnionitis
Source: Sci Rep. 2022 Apr 20;12:6537. doi: 10.1038/s41598-022-10258-0 (PMC9021264; doi:10.1038/s41598-022-10258-0)

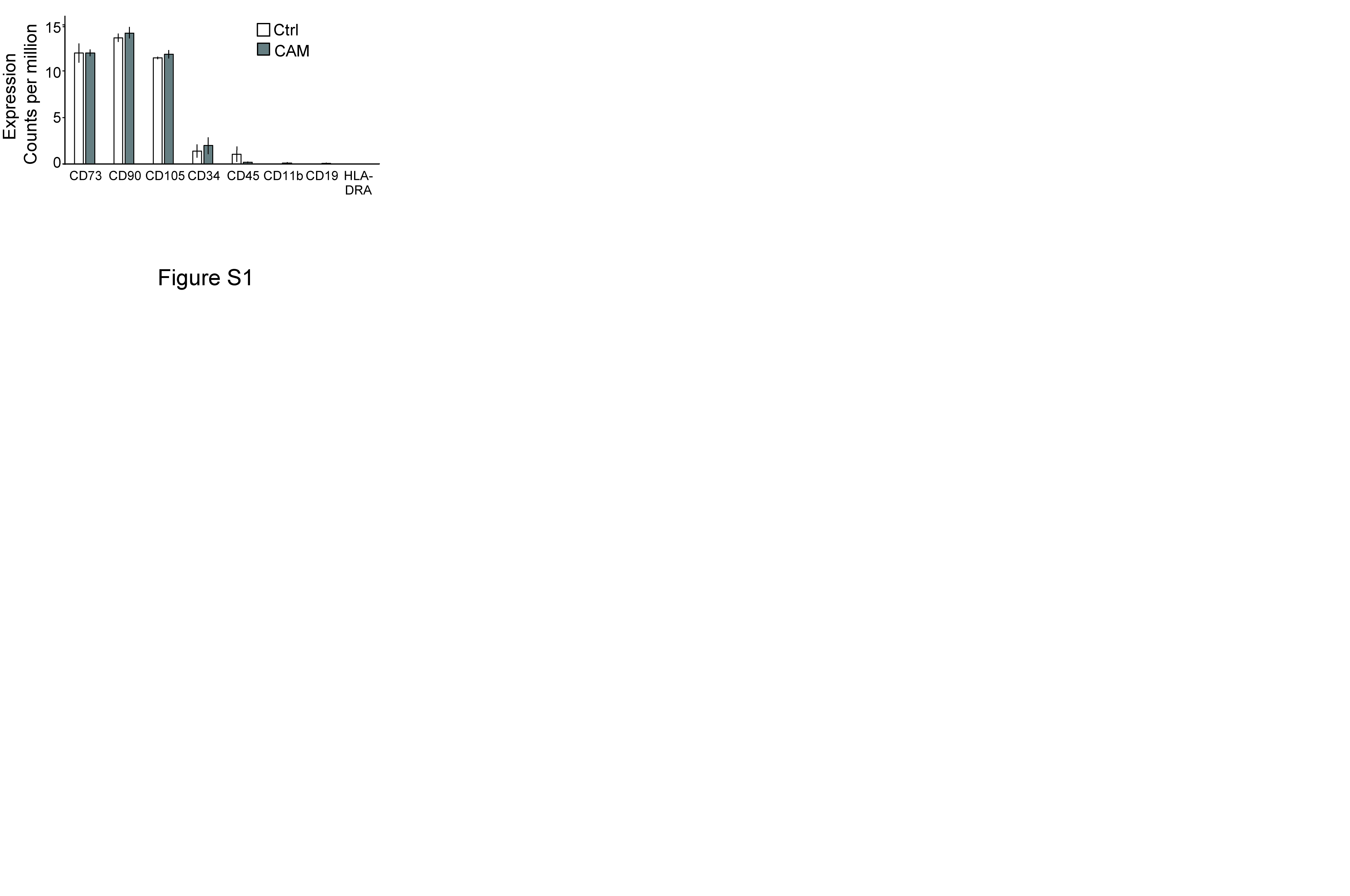

Supplement: Supplementary file 2 — Supplementary Figure S1. [file 41598_2022_10258_MOESM2_ESM.tif]
