# Supplementary material for: Transcriptome analysis of umbilical cord mesenchymal stem cells revealed fetal programming due to chorioamnionitis
Source: Sci Rep. 2022 Apr 20;12:6537. doi: 10.1038/s41598-022-10258-0 (PMC9021264; doi:10.1038/s41598-022-10258-0)
Supplement: Supplementary file 3 — Supplementary Table S1. [file 41598_2022_10258_MOESM3_ESM.docx]

**Supplementary Table S1 Clinical background of subjects**

| Sample | Gestational  age (weeks) | Birth  weight (g) | Sex | Apgar  score  1/5 min | Parity | Perinatal  history | Maternal  complication | Chorioamnionitis (pathological grade) | Funisitis  (pathological grade) | CLD | Development at 1.5y | Development at 3y | RNAseq |
| --- | --- | --- | --- | --- | --- | --- | --- | --- | --- | --- | --- | --- | --- |
| Control 1 | 25 | 802 | Male | 5 / 7 | 0 | active premature labor | - | 1 | 0 | Yes | border | retardation | ○ |
| Control 2 | 29 | 1080 | Male | 3 / 7 | 0 | non-reassuring fetal status | HDP* | 0 | 0 | No | Normal | border | ○ |
| Control 3 | 30 | 1344 | Female | 6 / 8 | 3 | active premature labor | - | 0 | 0 | No | No data | No data | ○ |
| Control 4 | 28 | 914 | Female | 6 / 8 | 0 | maternal  heart failure | HDP* | 0 | 0 | Yes | No data | No data |  |
| Control 5 | 28 | 1140 | Female | 5 / 6 | 1 | abruption  of placenta | - | 1 | 0 | No | Border | No data |  |
| CAM 1 | 26 | 1016 | Male | 6 / 8 | 1 | active premature labor | GDM† | 3 | 0 | Yes | retardation | retardation | ○ |
| CAM 2 | 29 | 1290 | Female | 6 / 8 | 0 | active premature labor | - | 3 | 3 | No | border | Border | ○ |
| CAM 3 | 25 | 936 | Female | 1 / 6 | 0 | active premature labor | - | 3 | 3 | Yes | normal | No data | ○ |
| CAM 4 | 29 | 1385 | Male | 8 / 8 | 0 | active premature labor | - | 2 | 0 | No | normal | normal |  |

*: HDP: Hypertensive Disorders of Pregnancy †: GDM: Gestational Diabetes Mellitus
