# Supplementary material for: Transcriptome analysis of umbilical cord mesenchymal stem cells revealed fetal programming due to chorioamnionitis
Source: Sci Rep. 2022 Apr 20;12:6537. doi: 10.1038/s41598-022-10258-0 (PMC9021264; doi:10.1038/s41598-022-10258-0)
Supplement: Supplementary file 4 — Supplementary Table S2. [file 41598_2022_10258_MOESM4_ESM.docx]

**Supplementary table S2 Primers used for real-time PCR**

| Gene | Forward primer | Reverse primer |
| --- | --- | --- |
| TGF-β1 | ACTACTACGCCAAGGAGGTCAC | TGCTTGAACTTGTCATAGATTTCG |
| CTGF | CTTGCGAAGCTGACCTGGAA | AGCTCAAACTTGATAGGCTTGGAGA |
| αSMA | CTATGCCTCTGGACGCACAAC | CCCATCAGGCAACTCGTAACTC |
| GAPDH | ACCACAGTCCATGCCATCAC | TCCACCACCCTGTTGCTGTA |
